# Examining Sleep‐Related Problems in Youth With Misophonia

**DOI:** 10.1002/jclp.70075

**Published:** 2025-12-07

**Authors:** Kevin M. Wagner, Matti Cervin, Catherine E. Rast, Mered Parnes, Nicholas Murphy, Samuel Spencer, Eric A. Storch, Andrew G. Guzick

**Affiliations:** ^1^ Menninger Department of Psychiatry & Behavioral Sciences Baylor College of Medicine Houston TX USA; ^2^ Department of Clinical Sciences Lund University Lund Sweden; ^3^ Department of Psychiatry and Behavioral Sciences University of Pennsylvania Philadelphia PA USA

**Keywords:** adolescents, anxiety, misophonia, sleep, youth

## Abstract

The relationship between misophonia and sleep‐related problems (SRPs) in youth is underexplored. This paucity of research is concerning because SRPs might be elevated among youth with misophonia. If left untreated, SRPs can contribute to long‐term health consequences. Thus, in this study we examined the link between misophonia and SRPs in youth aged 8 to 17. In this study, we compared SRPs in 102 children and adolescents with clinically significant misophonia to SRPs in a normative youth sample and a sample of 94 youth with anxiety disorders. We also examined the extent to which SRPs were associated with misophonia severity. Approximately 30% of youth with misophonia endorsed clinical levels of SRPs. SRPs were more prevalent in youth with misophonia compared to normative data from the general youth population (*d* = 1.22) and similar to SRPs in youth with anxiety disorders (*d* = 0.13). Youth with more severe misophonia symptoms had greater difficulties with SRPs and this association was attenuated but still significant when adjusting for gender, age, and internalizing and externalizing symptoms. In this study, we provided the first evidence of substantial issues with SRPs in youth with misophonia. Importantly, there was a moderate association between misophonia severity and SRPs, indicating that SRPs should be carefully assessed and potentially addressed in treatment for youth with misophonia. We discussed suggestions for clinical practice and future research.

Misophonia is characterized by strong physiological and emotional reactions (e.g., anger, anxiety, disgust) in response to specific trigger sounds and related visual stimuli and can lead to significant distress and impairment (Swedo et al. 2022). Emerging research on misophonia has provided important insights into this condition. First, it has become clear that co‐occurring mental health problems are common among youth with misophonia (Guzick et al. 2023; Siepsiak et al. 2025), with 79% meeting criteria for a psychiatric disorder among a sample of 102 youth with clinically significant misophonia (Guzick et al.). Furthermore, recent studies have suggested important roles of sound sensitivities, learning, and emotion regulation as etiological pathways in misophonia (Rosenthal et al. 2023a). Despite this growing body of research, a critical aspect that remains underexplored is the relation between misophonia and sleep‐related problems (SRPs), particularly among youth.

On a more general basis, the paucity of research concerning youth with misophonia is a clear limitation, as misophonia typically begins in childhood or adolescence (Potgieter et al. 2019). In three independent samples, internalizing mental health problems (e.g., symptoms related to anxiety and depressive disorders) have been found to be elevated among youth with misophonia, including one study comparing 42 youth with clinically significant misophonia to 35 without (Siepsiak et al. 2025), one study comparing 102 youth with clinically significant misophonia to population norms (Guzick et al. 2023), and a study comparing randomly sampled youth with elevated misophonia symptoms to those without (Rinaldi et al. 2022). These elevations are notable because internalizing symptoms can contribute to SRPs (Tarokh et al. 2016). Given the importance of early intervention for preventing negative long‐term consequences (McGorry and Mei 2018), misophonia and associated SRPs in youth may continue to be impairing into adulthood if left untreated.

Broadly, SRPs encompass an array of challenges, including difficulties with falling asleep, staying asleep, and achieving restorative sleep (Bevans et al. 2019). These difficulties are associated with a range of significant mental and physical health consequences (Bevans et al. 2019). Thus, identifying and addressing sleep‐related problems in youth with misophonia may offer a valuable point of clinical intervention. There are several reasons why SRPs might be prevalent among youth with misophonia. First, it is possible that noise triggers could cause disruption in sleep patterns. While exposure to noise at nighttime can lead to SRPs in individuals without misophonia (Halperin 2014), those with misophonia may have a heightened sensitivity to trigger sounds (Savard et al. 2022). Thus, youth with misophonia who are exposed to triggering stimuli at night (e.g., snoring, voices) could potentially experience increased SRPs.

Second, misophonia has been linked with an array of internalizing mental health conditions (e.g., depression and anxiety; Guzick et al. 2023; Rosenthal et al. 2023a; Siepsiak et al. 2025), which are known to be associated with SRPs (Orchard et al. 2020). Although one large study of adult patient with misophonia found low overall psychiatric comorbidity (e.g., 9% comorbid anxiety disorders; Jager et al. 2020), a number of other studies with youth in particular have reported higher psychiatric comorbidity rates (Guzick et al. 2023; Rosenthal et al. 2023a; Siepsiak et al. 2022). In the only study that carefully characterized comorbidity in youth with clinically significant misophonia, Guzick et al. (2023) found significant rates of interview‐confirmed psychological disorders (e.g., 56% had a comorbid anxiety disorder; 46% had a history of a depressive disorder). The present study aims to contribute to the ongoing research on misophonia and its link with factors important to mental health, namely SRPs.

In contrast to the misophonia literature, there is a well‐established body of research supporting a bidirectional association between anxiety and SRPs in youth (Brown et al. 2018; Willis and Gregory 2015). Further, internalizing symptoms (a broader concept including problems associated with negative affect), which may be elevated in youth with misophonia (Guzick et al. 2023; Rinaldi et al. 2022; Siepsiak et al. 2025), can disrupt circadian rhythms and sleep initiation and maintenance (Boyce and Barriball 2020; Kalmbach et al. 2018).

Third, case reports and clinical experience suggest a link between misophonia and SRPs. More specifically, in one case study, Dozier (2015) reported a case where a child experienced SRPs due to hearing breathing sounds from his brother in a shared bedroom. In another report, Sharan and Sharma (2020) described an adult misophonia case who had a history of SRPs during family trips due to snoring and breathing sounds from his parents. Similarly, Jager et al. (2022) reported an adult misophonia case where a patient had SRPs due to hyper‐focusing on her husband's snoring (a misophonic trigger sound), which prompted her to sleep alone to avoid this trigger. Additionally, Neal and Cavanna (2012) reported an adult misophonia case who had a history of SRPs in childhood. While data from case reports require interpretation with caution due to the uncontrolled nature of such studies, these examples highlight the significant impact that misophonia can have with regard to SRPs.

Youth is a developmental phase characterized by significant biopsychosocial changes (Christie and Viner 2005) and offers a valuable context for investigating SRPs and their association with misophonia (Trosman and Ivanenko 2021). The biopsychosocial changes characteristic of the transition from childhood to adolescence, such as the onset of puberty, cognitive development, and the establishment of social autonomy, can disrupt sleep‐wake patterns, making youth particularly susceptible to SRPs (Lucien et al. 2021).

Examining the association between SRPs and misophonia in youth is important given the rise in SRPs among youth populations more broadly (Sharma et al. 2021). For example, a meta‐analysis including 690,747 youth found a gradual and consistent decrease in sleep duration in this age group over the past century (Matricciani et al. 2012). More recently, data collected from the COVID‐19 pandemic suggested a rise in SRPs in youth (Dayton et al. 2022). Examinations of how frequent SRPs are in youth with misophonia can provide a more detailed understanding of this condition and help determine whether having misophonia is related to SRPs. This knowledge can be valuable in shaping treatment approaches for youth with misophonia, especially when misophonia co‐occurs with SRPs.

## 1 Current Study

Using data from a previously completed deep phenotyping study of youth with misophonia (Guzick et al. 2023), the first research aim was to compare SRPs in individuals with misophonia and individuals with anxiety (a group known to have elevated SRPs; Peterman et al. 2015) to normative scores from the general population. We hypothesized that those in the misophonia group and those in the anxiety group would have higher SRPs compared to normative data from the general youth population. We then examined SRPs among three subgroups: participants with only a diagnosis of misophonia, participants with only a diagnosis of anxiety, and participants with both a misophonia and anxiety diagnosis. Due to a cumulative effect, we hypothesized that participants with co‐occurring misophonia and anxiety diagnoses would have higher levels of SRPs compared to participants with only a single diagnosis of either misophonia or anxiety.

The second research aim was to examine the extent to which severity of misophonia was related to SRPs. We hypothesized that higher levels of misophonia severity would be significantly associated with higher levels of SRPs even when statistically controlling for gender, age, and internalizing and externalizing psychopathology.

## 2 Methods

### 2.1 Participants

Analyses for the present study included a total of 196 youth, with 102 in the misophonia group and 94 in the anxiety group. In the full sample, the average age was 13.1 (SD = 2.6) and ages ranged from 8 to 17. The average age in the misophonia group was 13.7 years (SD = 2.5) and it was 12.4 years (SD = 2.6) in the anxiety group. In the misophonia sample, 30 youth self‐identified as male, 69 self‐identified as female, 1 self‐identified as trans male, 1 self‐identified as trans female, and 2 self‐identified as “Other.” In the anxiety group, 32 self‐identified as male, 54 self‐identified as female, and 8 self‐identified as “Other.” Because many participants with misophonia also met criteria for an anxiety disorder, for some analyses each participant was further classified into one of three subgroups: misophonia and no anxiety disorder (*n* = 45), misophonia and co‐occurring anxiety disorder (*n* = 57), and anxiety disorder and no misophonia (*n* = 94).

### 2.2 Procedures

In the present study, we analyzed an existing dataset on pediatric misophonia and anxiety disorders (Guzick et al. 2023). In the original study, participants with suspected misophonia or a suspected anxiety disorder were recruited for an initial screening interview. These participants were recruited through various online channels, such as professional clinical networks and social media sites dedicated to individuals with misophonia or anxiety. Eligibility criteria for the screening included participants and their parents providing consent or assent through an initial telehealth meeting. Additionally, both groups had to meet the following criteria: (1) child age between 8 and 17 years, (2) parental willingness to take part in the study, and (3) proficiency in English. The misophonia group had the unique prerequisite of having moderate levels of misophonia severity, indicated by a score of at least 10 on the Amsterdam Misophonia Scale (A‐MISO‐S; Schröder et al. 2013), as described in our prior work (Guzick et al. 2023; Cervin et al. 2023). The anxiety group had the unique prerequisites of scoring a *t*‐score of at least 60 on the Multidimensional Anxiety Scale for Children (MASC; March and Parker 2014), receiving a diagnosis of an anxiety disorder according to the Mini International Neuropsychiatric Interview for Children and Adolescents (MINI‐KID; Sheehan et al. 2010), and having a score of ≤ 4 on the A‐MISO‐S (Schröder et al. 2013), representing minimal misophonia symptoms.

The participants engaged in a separate assessment conducted via a telehealth platform, where they completed online questionnaires and underwent a clinical interview, including the Misophonia Assessment Interview (MAI; Lewin et al. 2021) and the MINI‐KID (Sheehan et al. 2010). Interviewers, comprised of research coordinators and doctoral students in psychology, received extensive training to ensure the assessments' validity and reliability under the supervision of a licensed psychologist. Initially, the study recruited 112 youth with suspected misophonia and 140 with suspected anxiety disorders. Afterwards, 102 youth from the misophonia group and 94 from the anxiety group were retained in the final sample. Exclusions in the misophonia group were primarily due to mild misophonia symptoms or scheduling challenges (Guzick et al. 2023). Exclusions in the anxiety group were related to low MASC scores, high A‐MISO‐S scores, the absence of an anxiety disorder diagnosis, or difficulties in scheduling assessment visits (Guzick et al.). The study, conducted virtually, received approval from the Institutional Review Board at Baylor College of Medicine.

The data used in this study are not publicly available due to their sensitive nature. Data sharing for some of the variables may be possible depending on ethical approval. Further enquiries can be directed to the corresponding author.

### 2.3 Measures

#### 2.3.1 Misophonia Severity

We used the Amsterdam Misophonia Scale (A‐MISO‐S) to assess misophonia severity in children, which consists of six Likert items measuring various aspects of misophonia (Schröder et al. 2013). Two sample items include “How much of your time is occupied by misophonic sounds?” and “How much do these misophonic sounds interfere with your social, work or role functioning?”. The A‐MISO‐S was administered in a self‐report format in the present study. The measure has demonstrated good psychometric properties in youth with misophonia (Cervin et al. 2023). Of note, it is recommended to exclude item 4 from the total score because the measure exhibits better internal consistency when this item is omitted (Cervin et al. 2023). In our study, the internal consistency was *α* = 0.68 with all six items but improved to *α* = 0.76 when using the five‐item version. Throughout the present study, we used the five‐item measure.

As an additional measure of misophonia severity, we used the Misophonia Assessment Questionnaire (MAQ; Johnson and Dozier 2013). Specifically, the child‐report (C‐MAQ) and parent‐report (P‐MAQ) versions of the MAQ were used. Two items from the C‐MAQ include “My sound issues currently make me unhappy” and “My sound issues currently create problems for me”. The P‐MAQ has similar items as the C‐MAQ, but the stem “My sound issues…” is replaced with “I feel that my child's sound issues…”. These versions of the MAQ each include 21 Likert items, and have demonstrated good psychometric properties when assessing misophonia severity in youth (Cervin et al. 2023). Both measures displayed high internal consistency in the present study, with *α* = 0.94 for the C‐MAQ and *α* = 0.93 for the P‐MAQ.

Of note, including three measures of misophonia severity allowed us to examine the robustness of findings across different informants and measurement formats. The A‐MISO‐S is a brief child‐report measure focused on functional interference and symptom severity, the C‐MAQ is a broader child‐report capturing subjective distress and life impairment, and the P‐MAQ offers a complementary parent‐report perspective. Together, these measures provided a more comprehensive understanding of misophonia severity in youth.

#### 2.3.2 SRPs

We used the 8‐item parent proxy of the Patient‐Reported Outcomes Measurement Information System Sleep Disturbance Short Form (PROMIS‐SD), which is a parent‐report of SRPs in the child over the past 7 days (Health Measures 2022). The PROMIS‐SD is a well‐established tool for assessing SRPs in youth and the scoring manual includes *t*‐scores that are standardized such that a score of 50 reflect the average for the U.S. general population (Health Measures). Using data from 1477 parents who had children aged 5 to 17, the PROMIS‐SD was post‐stratified to match U.S. census benchmarks for age, gender, region, metro status, and household income (Forrest et al. 2018). The raw scores are aggregated into a single total score, where higher scores represent more SRPs. We used established norms to convert the summed scores into a *t*‐score. In the present study, Cronbach's alpha for the PROMIS‐SD SRP measure was *α* = 0.91.

#### 2.3.3 Externalizing and Internalizing Symptoms

Using the Child Behavior Checklist (CBCL; Achenbach and Rescorla 2001), we created the externalizing composite variable by summing the scores from the rule‐breaking and aggressive behaviors subscales, as outlined in the CBCL manual (Achenbach and Rescorla). In the present study, Cronbach's alpha for the externalizing composite variable was *α* = 0.89. We used the CBCL's anxious/depressed subscale as a measure of internalizing problems, which had an alpha level of *α* = 0.87 in the present study. We decided against creating the CBCL's internalizing composite variable due to its inclusion of the somatic subscale, which contains two SRP items (Achenbach and Rescorla 2001).

### 2.4 Statistical Analysis

We conducted all analyses in R Statistical Software version 4.3.2 (R Core Team 2023). For the first aim, which sought to compare the frequency of SRPs across groups, we performed two one‐sample *t*‐tests to assess whether the misophonia group's and the anxiety group's mean *t*‐scores on the PROMIS‐SD measure were statistically different from the normative mean of 50 (Health Measures 2022). For these two *t*‐tests, the assumption of independence was inherently met because we compared our sample to a normative mean of 50. A visual inspection of histograms and Q‐Q plots supported the assumption of normality for the *t*‐tests. The assumption of homogeneity of variances was not applicable due to comparing our sample to a fixed value of 50 (Health Measures 2022). In line with this first aim, we also examined whether the frequency of SRPs differed between the misophonia and anxiety groups. We then computed sensitivity analyses (Welch's ANOVA) to examine whether the prevalence of SRPs differed among the three subgroups (i.e., youth with misophonia but not anxiety, youth with anxiety but not misophonia, and youth with anxiety and misophonia). With respect to the assumption of homogeneity of variances, the Brown‐Forsythe test suggested that there was not enough evidence to conclude that there were statistically significant differences in variances across the three subgroups (*p* = 0.14).

For the second aim, which was to examine the extent to which misophonia severity was related to SRPs, we performed a series of regression analyses. Each regression model included SRPs as the dependent variable and one of the three measures of misophonia severity as an independent variable: C‐MAQ, P‐MAQ, and A‐MISO‐S. Additional covariates (i.e., age, gender, and externalizing and internalizing problems) were included in follow‐up analyses. Gender was dichotomized as male vs non‐male, and the other covariates were continuous variables. Of note, for the binary gender variable, the “male” category included youth who self‐identified as male or trans male, while the “non‐male” category included youth who self‐identified as female, trans female, or “other.” A visual inspection of scatterplots and Q‐Q plots supported the assumptions of linearity, normality of residuals, and homoscedasticity for all regression models.

## 3 Results

### 3.1 SRPs in Youth with Misophonia

**Figure 1:**
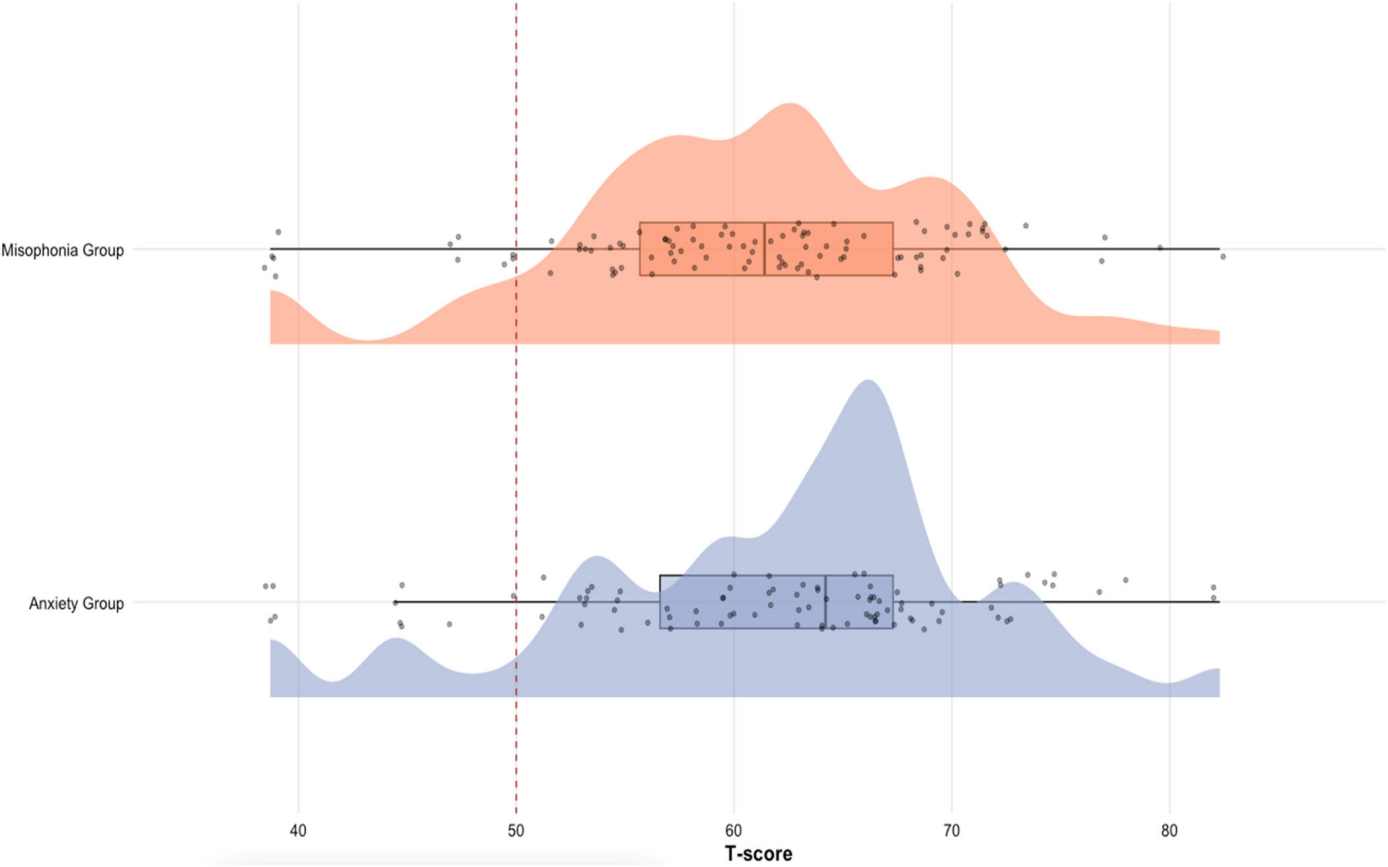
Distribution of sleep disturbance *t*‐scores in youth with misophonia and anxiety. *Note*. Figure 1 presents the distribution of PROMIS Sleep Disturbance *t*‐scores for youth with misophonia and anxiety. The figure includes: density plots to visualize score distributions, boxplots summarizing group medians and interquartile ranges, jittered raw data points representing individual participants, and a vertical dashed red line indicating the normative *t*‐score of 50 (Health Measures 2022) to highlight deviation from the population mean. Both groups demonstrated elevated sleep disturbance relative to the normative mean. This visualization complements the statistical analyses by highlighting the clinical significance of group differences. The figure was created in R using the *ggplot2* (Wickham 2016) and *ggdist* (Kay 2024) packages.

The first research aim was to compare SRPs in individuals in the misophonia group (*n* = 102) and the anxiety group (*n* = 94) to normative scores from the general population and with each other, using the PROMIS‐SD measure. A Bonferroni correction was applied to account for the following three *t*‐tests, yielding a corrected alpha of 0.017 (Armstrong 2014). For the misophonia group, a one‐sample *t*‐test revealed that the mean *t*‐score for the misophonia group (M = 60.70, SD = 8.79) was significantly higher than the normative mean of 50 (*t* = 12.18, *df* = 99, *p* < 0.001, 95% CI: 58.96–62.45) with a large effect size (*d* = 1.22). In the misophonia group, 31 participants (30%) had *t‐*scores of 65 or higher. For the anxiety group, a one‐sample *t*‐test revealed that the mean *t*‐score for the anxiety group (*M* = 61.91, SD = 9.5) was significantly higher than the normative mean of 50 (*t* = 11.96, *df* = 90, *p* < 0.001, 95% CI: 59.93–63.89) with a large effect size (*d* = 1.25). In the anxiety group, 40 participants (43%) had *t‐*scores of 65 or higher. Additionally, an independent samples *t*‐test revealed that the mean *t*‐score for the misophonia group was not statistically significantly different than the mean *t*‐score for the anxiety group: *t* = 0.91, *df* = 183.58, *p* = 0.365, 95% CI: −1.42–3.82 with a negligible effect size (*d* = 0.13). Figure 1 visualizes the distribution of sleep disturbance *t*‐scores across youth with misophonia and anxiety, illustrating elevated scores in both groups relative to the normative mean of 50 (Health Measures 2022). These findings supported the hypotheses that youth in the misophonia group and youth in the anxiety group experienced heightened levels of SRPs compared to the normative mean.

We then computed sensitivity analyses to examine SRPs in youth across the three subgroups: “misophonia and no anxiety disorder,” “misophonia and co‐occurring anxiety disorder,” and “anxiety disorder and no misophonia” by comparing these three groups to each other with a Welch's ANOVA. The *t*‐scores in all three subgroups were statistically different than the normative mean of 50 (see Table 1). Results from a Welch's ANOVA did not support the hypotheses that there would be statistically significant differences in SRPs between the three groups: *F*(2,105.98) = 2.0212, *p* = 0.1376.

**Table 1:** *T‐*Scores for Each Subgroup Relative to the Normative Mean of 50.

Subgroup	*M*	SD	*t*	*df*	*p* value	95% CI
Misophonia and no anxiety disorder (*n* = 45)	58.90	9.6	6.52	44	< 0.001	56.15–61.66
Misophonia and co‐occurring anxiety disorder (*n* = 57)	62.17	8.27	10.92	54	< 0.001	59.94–64.41
Pooled misophonia group (*n* = 112)	60.70	8.79	12.18	99	< 0.001	58.96–62.45
Anxiety disorder and no misophonia (*n* = 94)	61.91	9.5	11.96	90	< 0.001	59.9–63.89

### 3.2 Factors Associated with SRPs in Youth with Misophonia

The second aim was to examine the association between misophonia severity and SRPs in the misophonia group by employing regression analysis. We controlled for age, self‐reported gender, and internalizing and externalizing problems in each of the following three regression models. Misophonia severity was a significant independent variable of SRPs across all three measures. Specifically, A‐MISO‐S scores predicted SRPs (*β* = 0.19, *t* (94) = 1.99, *p* = 0.049, *r*² = 0.19), as did C‐MAQ scores (*β* = 0.22, *t* (94) = 2.11, *p* = 0.037, *r*² = 0.20), and P‐MAQ scores (*β* = 0.24, *t* (93) = 2.44, *p* = 0.017, *r*² = 0.21). Overall, results from these regression analyses suggested that misophonia severity was significantly linked with SRPs above and beyond what was accounted for age, gender, and internalizing and externalizing problems.

## 4 Discussion

This is the first study to specifically examine SRPs in youth with misophonia. When compared to youth from the general population, and in line with our hypothesis, SRPs were much more frequent in youth with misophonia and 30% of the misophonia group reported clearly elevated scores with *t‐*scores of 65 or higher. When compared to youth with anxiety disorders, similar levels of SRPs emerged and there was no statistically significant difference between the two groups. We had hypothesized that youth dually affected by misophonia and anxiety disorders would have the most SRPs. This hypothesis was not confirmed, as SRPs did not significantly differ among youth with misophonia only, anxiety only, and the combination. As for misophonia specifically, the ANOVA revealed that youth with misophonia, even those without a comorbid anxiety disorder, had significantly elevated SRPs relative to the normative mean. This pattern suggests that misophonia itself, independent of anxiety, may contribute meaningfully to sleep disturbance. One important takeaway from these analyses is that misophonia may be a vulnerability factor for SRPs in youth, consistent with case reports (Dozier 2015; Jager et al. 2022; Neal and Cavanna 2012; Sharan and Sharma 2020).

This study also examined the link between SRPs and misophonia severity in the misophonia group using three different measure of misophonia severity, the A‐MISO‐S and the child‐ and parent‐reported MAQ (Johnson and Dozier 2013). Across measures, we found that SRPs were associated with more severe misophonia. Notably, relations remained statistically significant when accounting for other factors like age, gender, and internalizing and externalizing symptoms—all of which have been related to SRPs in prior literature (Liu et al. 2022; Williamson et al. 2021), although the association for A‐MISO‐S only trended towards significance after adjusting for the covariates. However, the R‐squared values in the regression models (ranging from 0.07 to 0.21) suggest a significant portion of unexplained variance in the relationship between SRPs and misophonia severity, indicating that SRPs are likely one of many factors associated with misophonia severity.

The implications of our findings carry weight in the realm of clinical practice, particularly in understanding and addressing SRPs in youth with misophonia. The heightened levels of SRPs observed in this study suggest that healthcare professionals should consider incorporating sleep assessments into the evaluation and treatment of misophonia in youth populations—or at the very least, inquire about it directly during clinical intakes (Rosenthal et al. 2023b). Consistent with our findings, clinicians should be aware that misophonia may serve as a vulnerability factor for SRPs in youth. Moreover, given the independent association between misophonia severity and SRPs, treatment plans for youth with misophonia should take into account the potential impact of misophonia on sleep, such as common misophonic trigger sounds (Guzick et al. 2023). Behavioral treatments for insomnia have been shown to have clear downstream positive impacts on anxiety, depression, and other metrices of emotional health (Mirchandaney et al. 2022). It is possible that the association between misophonia and sleep is also bidirectional; sensitivity to triggers at night may disrupt sleep, which may in turn inhibit adaptive emotion regulation the next day and lead to more sensitivity to triggers. If this is the case, helping youth achieve better sleep using evidence‐based strategies may also improve their ability to cope with misophonic triggers. However, the cross‐sectional nature of the present study precludes us from drawing causal inferences concerning these relationships. Thus, additional research using temporally dense longitudinal methods are needed to further examine the link between SRPs and misophonia symptomology.

Given the bidirectional link between sleep quality and mental health and emotion regulation (Bai et al. 2020; Brown et al. 2018), our results underscored the importance of early identification and targeting of sleep disturbances in youth with misophonia. The potential neurobiological mechanisms underlying this association, although not within the scope of this study, could involve the intricate interplay between misophonic triggers, heightened emotional responses, and disruptions in neural circuits regulating sleep (Stewart et al. 2020). Further exploration of these neurobiological aspects could enhance our understanding of the pathophysiology of misophonia, paving the way for more targeted interventions (Neacsiu et al. 2022).

We acknowledge several limitations in interpreting results from the present findings. First, as mentioned previously, the cross‐sectional design of the research limits our ability to establish causal relations between misophonia and SRPs. Longitudinal studies would provide a more robust understanding of the temporal dynamics and potential bidirectional influences between misophonia severity and SRPs over time (Vazsonyi et al. 2022). Second, the reliance on self‐report and observer‐report measures, including the PROMIS‐SD measure for SRPs and the misophonia severity assessments, introduces the possibility of response and recall bias (van de Mortel 2008). Future research would benefit from incorporating other measures, such as actigraphy or polysomnography, to assess sleep patterns more objectively (van Meter and Anderson 2020). Another limitation is that we relied on the parent's report of the child's SRPs because child‐reported SRPs were not collected in the study. While this parent‐report is helpful because parents often manage their child's sleep routines, it may not capture the child's subjective experiences, such as difficulties falling asleep that are unreported to the parent. Future studies would benefit from including child self‐reported SRPs the parent's report. Similarly, although a structured diagnostic interview was conducted (Sheehan et al. 2010), we did not include detailed diagnostic data beyond our primary focus on misophonia and anxiety, which is reported elsewhere (Guzick et al. 2023). This limits our ability to isolate the impact of other specific co‐occurring disorders (e.g., major depressive disorder), though internalizing and externalizing symptoms were accounted for dimensionally in the analyses.

Additionally, this study focused on youth, and expanding the investigation to different age groups could elucidate potential developmental variations in the relation between misophonia and SRPs (Wang et al. 2016). Likewise, our decision to dichotomize gender into male versus non‐male for analytic purposes may have obscured important differences among gender‐diverse youth. Future research should include sufficient representation of gender‐diverse groups to enable analyses that explore meaningful within‐group variation in misophonia severity and SRPs, rather than collapsing across gender identities. Further, the assessment of misophonia has advanced considerably since we began this study. Although the MAQ and A‐MISO‐S remain among the only validated assessment for youth with clinically significant misophonia (see also Rinaldi et al. 2022), more robust assessment of misophonia severity is needed in future work. Additionally, in this preliminary study, each misophonia severity measure was tested individually as a predictor of SRPs; however, larger confirmatory studies are warranted to replicate these findings. Lastly, the statistical norms used in the assessments were established before the COVID‐19 pandemic. The data collection took place between Fall 2020 and Fall 2021 (during the pandemic), potentially rendering the pre‐pandemic norms less applicable.

## Author Contributions

All authors contributed significantly to this manuscript. Kevin M. Wagner contributed to conceptualization, conducting formal analysis, and drafting the initial manuscript. Matti Cervin, Eric A. Storch, and Andrew G. Guzick contributed to the conceptual framework and provided input through thorough review and editing processes. Catherine E. Rast, Samuel Spencer, and Mered Parnes reviewed and edited the manuscript. Nicholas Murphy contributed to data collection and analysis. All authors reviewed and approved the final version of the manuscript.

## Data Availability

The data that support the findings of this study are available from the corresponding author upon reasonable request.

## References

[jclp70075-bib-0001] Achenbach, T. M. , and L. Rescorla 2001. Manual for the ASEBA School‐Age Forms & Profiles: An Integrated System of Multi‐Informant Assessment. ASEBA.

[jclp70075-bib-0003] Armstrong, R. A. 2014. “When to Use the Bonferroni Correction.” Ophthalmic and Physiological Optics 34, no. 5: 502–508. 10.1111/opo.12131. 24697967

[jclp70075-bib-0004] Bevans, K. B. , L. J. Meltzer , A. De La Motte , A. Kratchman , D. Viél , and C. B. Forrest . 2019. “Qualitative Development and Content Validation of the PROMIS Pediatric Sleep Health Items.” Behavioral Sleep Medicine 17, no. 5: 657–671. 29693445 10.1080/15402002.2018.1461102

[jclp70075-bib-0005] Boyce, P. , and E. Barriball . 2020. “Circadian Rhythms and Depression.” Australian Family Physician 39, no. 5: 307–310. 10.3316/informit.144294711037521. 20485718

[jclp70075-bib-0006] Brown, W. J. , A. K. Wilkerson , S. J. Boyd , D. Dewey , F. Mesa , and B. E. Bunnell . 2018. “A Review of Sleep Disturbance in Children and Adolescents with Anxiety.” Journal of Sleep Research 27, no. 3: e12635. 29193443 10.1111/jsr.12635

[jclp70075-bib-0007] Cervin, M. , A. G. Guzick , J. Clinger , et al. 2023. “Measuring Misophonia in Youth: A Psychometric Evaluation of Child and Parent Measures.” Journal of Affective Disorders 338: 180–186. 37263358 10.1016/j.jad.2023.05.093 PMC11165319

[jclp70075-bib-0008] Christie, D. , and R. Viner . 2005. “Adolescent Development.” BMJ 330, no. 7486: 301–304. 15695279 10.1136/bmj.330.7486.301 PMC548185

[jclp70075-bib-0009] Dayton, L. , X. Kong , T. W. Powell , et al. 2022. “Child Mental Health and Sleep Disturbances During the Early Months of the COVID‐19 Pandemic in the United States.” Family & Community Health 45, no. 4: 288–298. 35985027 10.1097/FCH.0000000000000338 PMC9394872

[jclp70075-bib-0010] Dozier, T. H. 2015. “Etiology, Composition, Development and Maintenance of Misophonia: A Conditioned Aversive Reflex Disorder.” Psychological Thought 8, no. 1: 114–129.

[jclp70075-bib-0011] Forrest, C. B. , L. J. Meltzer , C. L. Marcus , et al. 2018. “Development and Validation of the Promis Pediatric Sleep Disturbance and Sleep‐Related Impairment Item Banks.” Sleep 41, no. 6: zsy054. 10.1093/sleep/zsy054. 29546286

[jclp70075-bib-0013] Guzick, A. G. , M. Cervin , E. E. A. Smith , et al. 2023. “Clinical Characteristics, Impairment, and Psychiatric Morbidity in 102 Youth With Misophonia.” Journal of Affective Disorders 324: 395–402. 36584703 10.1016/j.jad.2022.12.083 PMC9878468

[jclp70075-bib-0014] Halperin, D. 2014. “Environmental Noise and Sleep Disturbances: A Threat to Health?” Sleep Science 7, no. 4: 209–212. 26483931 10.1016/j.slsci.2014.11.003 PMC4608916

[jclp70075-bib-0015] Health Measures . 2022. Sleep Scoring Manual. https://www.healthmeasures.net/images/PROMIS/manuals/Scoring_Manual_Only/PROMIS_Sleep_Scoring_Manual_05Dec2023.pdf.

[jclp70075-bib-0016] Jager, I. , P. De Koning , T. Bost , D. Denys , and N. Vulink . 2020. “Misophonia: Phenomenology, Comorbidity and Demographics in a Large Sample.” PLoS One 15, no. 4: e0231390. 32294104 10.1371/journal.pone.0231390 PMC7159231

[jclp70075-bib-0017] Jager, I. , N. Vulink , A. van Loon , et al. 2022. “Synopsis and Qualitative Evaluation of a Treatment Protocol to Guide Systemic Group‐Cognitive Behavioral Therapy for Misophonia.” Frontiers in Psychiatry 13: 794343. 10.3389/fpsyt.2022.794343. 35836662 PMC9275669

[jclp70075-bib-0019] Johnson, M. , and T. Dozier . 2013. Misophonia Assessment Questionnaire (MAQ). Revised by, edited by T. Dozier . Livermore, CA: Misophonia Institute.

[jclp70075-bib-0020] Kalmbach, D. , A. Cuamatzi‐Castelan , C. Tonnu , et al. 2018. “Hyperarousal and Sleep Reactivity in Insomnia: Current Insights.” Nature and Science of Sleep 10: 193–201. 10.2147/NSS.S138823 PMC6054324 30046255

[jclp70075-bib-0021] Kay, M. 2024. ggdist: Visualizations of distributions and uncertainty. R package version 3.3.2. 10.5281/zenodo.10782896. 37883271

[jclp70075-bib-0022] Lewin, A. B. , S. Dickinson , K. Kudryk , et al. 2021. “Transdiagnostic Cognitive Behavioral Therapy for Misophonia in Youth: Methods for a Clinical Trial and Four Pilot Cases.” Journal of Affective Disorders 291: 400–408. 10.1016/j.jad.2021.04.027. 34001373

[jclp70075-bib-0023] Lucien, J. N. , M. T. Ortega , and N. D. Shaw . 2021. “Sleep and Puberty.” Current Opinion in Endocrine and Metabolic Research 17, no. 7: 1–7. 35005296 10.1016/j.coemr.2020.09.009 PMC8730357

[jclp70075-bib-0024] March, J. S. , and J. D. Parker . 2014. “The Multidimensional Anxiety Scale for Children (MASC).” In The Use of Psychological TestING for Treatment Planning and Outcomes Assessment, 39–62. Routledge.

[jclp70075-bib-0025] Matricciani, L. , T. Olds , and J. Petkov . 2012. “In Search of Lost Sleep: Secular Trends in the Sleep Time of School‐Aged Children and Adolescents.” Sleep Medicine Reviews 16, no. 3: 203–211. 21612957 10.1016/j.smrv.2011.03.005

[jclp70075-bib-0026] McGorry, P. D. , and C. Mei . 2018. “Early Intervention in Youth Mental Health: Progress and Future Directions.” Evidence Based Mental Health 21, no. 4: 182–184. 30352884 10.1136/ebmental-2018-300060 PMC10270418

[jclp70075-bib-0027] van Meter, A. R. , and E. A. Anderson . 2020. “Evidence Base Update on Assessing Sleep in Youth.” Journal of Clinical Child and Adolescent Psychology 49, no. 6: 701–736. 33147074 10.1080/15374416.2020.1802735

[jclp70075-bib-0028] Mirchandaney, R. , R. Barete , and L. D. Asarnow . 2022. “Moderators of Cognitive Behavioral Treatment for Insomnia on Depression and Anxiety Outcomes.” Current Psychiatry Reports 24, no. 2: 121–128. 35061137 10.1007/s11920-022-01326-3 PMC8948126

[jclp70075-bib-0029] van de Mortel, T. F. 2008. “Faking It: Social Desirability Response Bias in Self‐Report Research.” Australian Journal of Advanced Nursing 25, no. 4: 40–48.

[jclp70075-bib-0030] Neacsiu, A. D. , V. Szymkiewicz , J. T. Galla , B. Li , Y. Kulkarni , and C. W. Spector . 2022. “The Neurobiology of Misophonia and Implications for Novel, Neuroscience‐Driven Interventions.” Frontiers in Neuroscience 16: 893903. 10.3389/fnins.2022.893903. 35958984 PMC9359080

[jclp70075-bib-0031] Neal, M. , and A. E. Cavanna . 2012. “P3 Selective Sound Sensitivity Syndrome (Misophonia) and Tourette Syndrome.” Journal of Neurology, Neurosurgery & Psychiatry 83, no. 10): e1–e1.

[jclp70075-bib-0032] Orchard, F. , A. M. Gregory , M. Gradisar , and S. Reynolds . 2020. “Self‐Reported Sleep Patterns and Quality Amongst Adolescents: Cross‐Sectional and Prospective Associations With Anxiety and Depression.” Journal of Child Psychology and Psychiatry 61, no. 10: 1126–1137. 32557672 10.1111/jcpp.13288

[jclp70075-bib-0033] Peterman, J. S. , M. M. Carper , and P. C. Kendall . 2015. “Anxiety Disorders and Comorbid Sleep Problems in School‐Aged Youth: Review and Future Research Directions.” Child Psychiatry and Human Development 46, no. 3: 376–392. 24962165 10.1007/s10578-014-0478-y

[jclp70075-bib-0034] Potgieter, I. , C. MacDonald , L. Partridge , R. Cima , J. Sheldrake , and D. J. Hoare . 2019. “Misophonia: A Scoping Review of Research.” Journal of Clinical Psychology 75, no. 7: 1203–1218. 30859581 10.1002/jclp.22771

[jclp70075-bib-0035] R Core Team . 2023. R: A Language and Environment for Statistical Computing. R Foundation for Statistical Computing.

[jclp70075-bib-0037] Rinaldi, L. J. , R. Smees , J. Ward , and J. Simner . 2022. “Poorer Well‐Being in Children With Misophonia: Evidence From the Sussex Misophonia Scale for Adolescents.” Frontiers in Psychology 13. 10.3389/fpsyg.2022.808379. PMC9019493 35465571

[jclp70075-bib-0038] Rosenthal, M. Z. , J. Campbell , and C. Altimus . 2023a. “Editorial: Advances in Understanding the Nature and Features of Misophonia.” Frontiers in Neuroscience 17. 10.3389/fnins.2023.1267682. PMC10523344 37771338

[jclp70075-bib-0039] Rosenthal, M. Z. , Y. Shan , and J. Trumbull . 2023b. “Treatment of Misophonia.” Advances in Psychiatry and Behavioral Health 3: 33–41. 10.1016/j.ypsc.2023.03.009.

[jclp70075-bib-0040] Savard, M.‐A. , A. G. Sares , E. B. J. Coffey , and M. L. D. Deroche . 2022. “Specificity of Affective Responses in Misophonia Depends on Trigger Identification.” Frontiers in Neuroscience 16: 879583. 35692416 10.3389/fnins.2022.879583 PMC9179422

[jclp70075-bib-0041] Schröder, A. , N. Vulink , and D. Denys . 2013. “Misophonia: Diagnostic Criteria for a New Psychiatric Disorder.” PLoS One 8, no. 1: e54706. 23372758 10.1371/journal.pone.0054706 PMC3553052

[jclp70075-bib-0042] Sharan, R. , and V. Sharma . 2020. “A Case of Bipolar Disorder and Misophonia.” The Primary Care Companion for CNS Disorders 22, no. 3: 26707. 10.4088/PCC.19l02523 32369688

[jclp70075-bib-0043] Sharma, M. , S. Aggarwal , P. Madaan , L. Saini , and M. Bhutani . 2021. “Impact of COVID‐19 Pandemic on Sleep in Children and Adolescents: A Systematic Review and Meta‐Analysis.” Sleep Medicine 84: 259–267. 34182354 10.1016/j.sleep.2021.06.002 PMC8687656

[jclp70075-bib-0044] Sheehan, D. V. , K. H. Sheehan , R. D. Shytle , et al. 2010. “Reliability and Validity of the Mini International Neuropsychiatric Interview for Children and Adolescents (MINI‐KID).” The Journal of Clinical Psychiatry 71, no. 3: 313–326. 20331933 10.4088/JCP.09m05305whi

[jclp70075-bib-0045] Siepsiak, M. , M. Z. Rosenthal , D. Raj‐Koziak , and W. Dragan . 2022. “Psychiatric and Audiologic Features of Misophonia: Use of a Clinical Control Group With Auditory Over‐Responsivity.” Journal of Psychosomatic Research 156: 110777. 35259551 10.1016/j.jpsychores.2022.110777

[jclp70075-bib-0054] Siepsiak, M ., A. Turek , M. Michałowska , M. Gambin , and W. Ł. Dragan . 2025. “Misophonia in Children and Adolescents: Age Differences, Risk Factors, Psychiatric and Psychological Correlates. A Pilot Study With Mothers' Involvement.” Child Psychiatry and Human Development 56, no. 3: 758–771. 37684420 10.1007/s10578-023-01593-y PMC12095346

[jclp70075-bib-0046] Stewart, E. M. , S. Landry , B. A. Edwards , and S. P. Drummond . 2020. “The Bidirectional Relationship Between Sleep and Health.” The Wiley Encyclopedia of Health Psychology: 165–188.

[jclp70075-bib-0047] Swedo, S. E. , D. M. Baguley , D. Denys , et al. 2022. “Consensus Definition of Misophonia: A Delphi Study.” Frontiers in Neuroscience 16: 841816. 10.3389/fnins.2022.841816. 35368272 PMC8969743

[jclp70075-bib-0048] Tarokh, L. , J. M. Saletin , and M. A. Carskadon . 2016. “Sleep in Adolescence: Physiology, Cognition and Mental Health.” Neuroscience and Biobehavioral Reviews 70: 182–188. 27531236 10.1016/j.neubiorev.2016.08.008 PMC5074885

[jclp70075-bib-0049] Trosman, I. , and A. Ivanenko . 2021. “Classification and Epidemiology of Sleep Disorders in Children and Adolescents.” Child and Adolescent Psychiatric Clinics of North America 30, no. 1: 47–64. 33223068 10.1016/j.chc.2020.08.002

[jclp70075-bib-0050] Vazsonyi, A. T. , D. Liu , and M. Blatny . 2022. “Longitudinal Bidirectional Effects Between Sleep Quality and Internalizing Problems.” Journal of Adolescence 94, no. 3: 448–461. 35390199 10.1002/jad.12039

[jclp70075-bib-0051] Wang, B. , C. Isensee , A. Becker , et al. 2016. “Developmental Trajectories of Sleep Problems From Childhood to Adolescence Both Predict and Are Predicted by Emotional and Behavioral Problems.” Frontiers in Psychology 7: 1874. 10.3389/fpsyg.2016.01874. PMC5131000 27990129

[jclp70075-bib-0052] Wickham, H. 2016. ggplot2: Elegant Graphics for Data Analysis (2nd ed. Springer. 10.1007/978-3-319-24277-4.

[jclp70075-bib-0053] Williamson, A. A. , N. Zendarski , K. Lange , et al. 2021. “Sleep Problems, Internalizing and Externalizing Symptoms, and Domains of Health‐Related Quality of Life: Bidirectional Associations From Early Childhood to Early Adolescence.” Sleep 44, no. 1: zsaa139. 32691073 10.1093/sleep/zsaa139 PMC7982136

[jclp70075-bib-0055] Willis, T. A. , and A. M. Gregory . 2015. “Anxiety Disorders and Sleep in Children and Adolescents.” Sleep Medicine Clinics 10, no.2: 125–131. 26055860 10.1016/j.jsmc.2015.02.002

